# Carbon Peel Laser Technique to Improve Skin Quality: Back to Science!

**DOI:** 10.5826/dpc.1004a113

**Published:** 2020-10-26

**Authors:** Stefania Guida, Elisabetta Fulgione, Ilaria D’Ambra, Graziella Babino, Giovanni Pellacani, Francesca Farnetani

**Affiliations:** 1 Dermatology Unit, Department of Surgical, Medical, Dental and Morphological Sciences related to Transplant, Oncology and Regenerative Medicine, University of Modena and Reggio Emilia, Modena, Italy; 2 Department of Dermatology, University of Campania Luigi Vanvitelli, Naples, Italy

**Keywords:** carbon peel laser, Q-switched laser, carbon suspension, enlarged pores

## Introduction

There is increasing interest in procedures to improve skin quality with short downtime. Specifically, topical carbon suspension combined with Q-switched Nd:YAG laser treatment (carbon peel laser technique) has gained popularity in the last few years. The aim of this case report is to present the use of the carbon peel laser in the treatment of a patient with enlarged skin pores and comedones.

## Case Presentation

We present herein the case of a 42-year-old woman who complained of oily skin, dilated pores, and noninflammatory comedones. The patient was treated with the carbon peel laser technique after acquiring the informed consent. Before treatment, the patient’s face was washed, and the carbon lotion (carbon pigments; Renlive Cosmeceuticals, Dueville, VI, Italy) was applied evenly over the face, except for the upper eyelids, eyebrows, and lips, and allowed to penetrate the skin and hair follicles for 10 minutes. The excess of carbon was removed and then the Q-switched 1064-nm Nd:YAG laser was employed to vaporize the skin surface. The Q-switched Nd:YAG laser (Xlase Plus, Biotec Italia srl, Dueville, VI, Italy) energy was delivered with a 7-mm diameter spot-sized handpiece at a repetition rate of 6 Hz. A single pass was carried out on face with the laser. The pulse width was of 9 nanoseconds and fluence was 2.6 J/cm2. Three sessions of treatment were performed at monthly intervals. Clinical pictures were taken before the laser sessions and 1 month after the last laser session.

**Figure 1:**
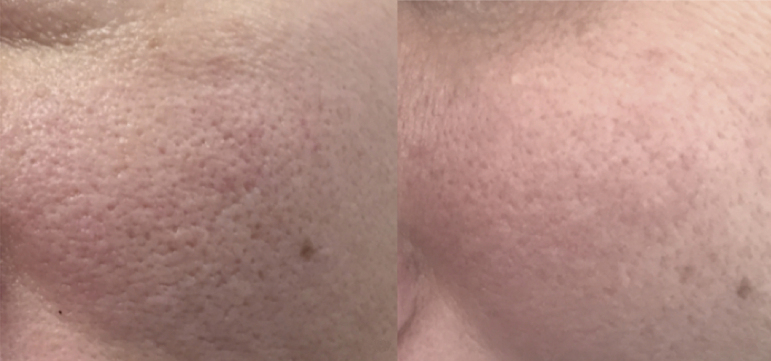
Clinical pictures of a 42-year-old woman (A) before and (B) after treatment with carbon peel laser.

Figure 1 shows clinical pictures before and after treatment, showing the improvement in the skin, mainly with a reduction of skin pore size. No severe adverse events were reported. Only a mild erythema after treatment was observed.

## Conclusions

The carbon peel laser technique was originally described in 1997 by Goldberg et al for laser hair removal. After providing the evidence for the effect on the hair follicle, the technique has been extended to treat acne vulgaris and pores [1,2].

In one study, different laser protocols were employed involving a small number of patients, and only a single experience for each indication was reported. Nevertheless, common mechanisms of action were hypothesized related to the peeling effect of the procedure, such as the reduction of microbial colonization of *P. acnes*, sebaceous gland function, together with the histologically proven thinning of the stratum corneum of the epidermis. Taken together, all these mechanisms might contribute to reduced inflammation [2].

Interestingly, the carbon peel laser technique has been proven to provide a peeling effect, cleaning off the skin surface and the plugged pores, thereby correcting the hypercornification of follicular epithelium that might block the physiologic outflow of sebum to the skin surface and reducing skin inflammation [2].

However, despite the popularity of this technique and much information accessible on the Internet, little scientific data are available on the literature. The last publication was reported in 2012. Our case report showed that treatment with carbon peel laser was effective for acne prone skin and had an excellent safety profile. Further studies are needed in order to find adequate protocols of treatment.
